# From the Americas to Southeast Asia: Navigating The Genomic Waves of Fall Armyworm (*Spodoptera frugiperda*) Invasions

**DOI:** 10.1111/eva.70139

**Published:** 2025-07-31

**Authors:** Maryam Nazir Chaudhary, Qasim Ayub, Wei Yee Wee, Shu Yong Lim, Fong Yoke Ling, Yan Eve Tan, Dilipkumar Masilamany, Beng‐Kah Song

**Affiliations:** ^1^ School of Science Monash University Malaysia Subang Jaya Selangor Darul Ehsan Malaysia; ^2^ Monash University Malaysia Genomics Platform (MUMGP) Subang Jaya Selangor Darul Ehsan Malaysia; ^3^ Rice Research Centre Malaysian Agricultural Research and Development Institute Kepala Batas Pulau Pinang Malaysia

**Keywords:** agricultural pest, evolutionary origins, invasive alien species, nuclear genome, population genomics, selective sweep

## Abstract

The fall armyworm (FAW), scientifically known as *Spodoptera frugiperda* , is an agricultural pest native to the American continents. Its larvae display voracious feeding behavior with a host range of over 350 plant species. The pest was first detected outside the Americas in 2016, subsequently spreading across Africa, Asia, and Oceania. As a country with substantial agricultural imports and exposure to regional migration routes, Malaysia presents a valuable case study for investigating the establishment and adaptation of invasive FAW populations. Forty‐two novel Malaysian FAW genomes were sequenced on the DNBSEQ‐G400 platform via DNBSeq. A subset of high‐quality genome‐wide single nucleotide polymorphisms was used to compare the evolution of both native and invasive FAW populations, with publicly available samples from another 18 countries from across the world. Our analyses revealed clear genetic differentiation between native and invasive FAW populations. We found little evidence to support West African populations as the founding source for Asian or East African invasions. Instead, Malaysian FAW clustered closely with populations from India, China, and East African countries, suggesting multiple, independent introductions into the region. Genomic outliers related to sensory perception, insecticide resistance, and heat tolerance were detected, likely contributing to the recent global success of FAW invasions. This study provides new genomic insights into the invasion history and adaptive strategies of FAW in Malaysia, contributing to a clearer picture of FAW movement across Asia and Africa. The results provide critical information for future pest management and policy‐making to mitigate the spread of this invasive pest.

## 1 Introduction

Invasive alien species (IAS) are responsible for vast economic losses stemming from factors such as reduced crop productivity (Diagne et al. 2021). Globally, insect IAS alone cost at least 70 billion USD for goods and services annually (Bradshaw et al. 2016). Effective control of IAS pests is crucial for safeguarding agroecosystems, but this effort is hampered by a limited understanding of the evolutionary mechanisms that contribute to the success of these pests (Chen and Schoville 2018). Recent advances in genomics, including next‐generation sequencing and population genomic analyses, offer new opportunities to explore the genetic makeup, migration patterns, and adaptation strategies of IAS (Lewald et al. 2021; Pélissié et al. 2018). Such information is invaluable for controlling further pest spread and influx of genetic variation. Additionally, studying the evolutionary responses of pests to diverse selection pressures (e.g., climate (Peng et al. 2023), insecticides (Cohen et al. 2022), host plant (Rêgo et al. 2020)) is essential for identifying sustainable pest control strategies.

One of the most destructive IAS in recent years is the fall armyworm (FAW), *Spodoptera frugiperda* (Lepidoptera: Noctuidae), a highly polyphagous pest native to tropical and subtropical regions of the Americas (Sparks 1979; Todd and Poole 1980). It displays voracious feeding behavior, with a wide host range of over 350 plant species (Deshmukh et al. 2021; Wan et al. 2021). Since its initial detection outside the Americas in West Africa in January 2016 (Cock et al. 2017; Goergen et al. 2016), the FAW has rapidly expanded across most of Africa (Feldmann et al. 2019), Asia (Sun et al. 2021) and Oceania (Rane et al. 2023). The FAW is responsible for significant economic losses, amounting to 9.4 billion USD in Africa alone (Eschen et al. 2021). In the Chinese provinces of Yunnan and Guangxi, the total costs of the FAW are estimated to be 830.51 and 346.09 million USD, respectively (Wu, Wu, et al. 2021). Beyond agricultural damage, the FAW also disrupts ecosystems by displacing native stemborers (Hailu et al. 2021), reducing predator diversity and attack intensity (ants, beetles, and spiders) (Rizali et al. 2021), and altering population densities of native species (Sokame et al. 2021).

Given its rapid rise as a global threat, elucidating the introduction pathways and genetic changes in FAW populations is critical for informing pest management strategies. The FAW's strong flight capabilities, with some individuals able to cover up to 1600 km in 30 h (Rose et al. 1975) and its migratory nature have been key to its success. Due to the absence of a diapause phase (Johnson 1987), the North American FAW undertakes seasonal migrations between southern Florida and Texas during winter and northern regions like Québec, Canada, in summer (Tessnow et al. 2023). However, FAW's swift colonization of Africa and Asia is unlikely to be solely explained by natural migration patterns, given the environmental and climatic barriers along the way (Nagoshi et al. 2018; Nagoshi et al. 2020). Thus, FAW dispersion across the globe was likely assisted by human transport and trade (Andrianto et al. 2024; Early et al. 2018; Rane et al. 2023; Tay, Rane, Padovan, et al. 2022), similar to other invasive agricultural pests (Paini et al. 2018). It is crucial to determine the evolutionary origins and gene flow avenues of the invasive FAW to prevent the proliferation of even more insecticide‐resistant strains.

Evidence indicates that the FAW was transported to non‐native lands before the official incidence reports of 2016. In 2014, the US Department of Agriculture (USDA) reported the interception of FAW larvae at US ports of entry from cargo originating from China, Indonesia, Israel, Micronesia, the Netherlands, Thailand, and Turkey (Gilligan and Passoa 2014; Kenis et al. 2023; Tay, Rane, Padovan, et al. 2022). Similarly, a total of 169 FAW larvae interceptions were recorded at the European border, dating back from 2003 to 2020 (Benjamin 2021). Additionally, previously unidentified samples from Indonesia, collected in 2015, were later confirmed as FAW, suggesting its possible introduction before its official documentation in 2019 (Andrianto et al. 2024). Moreover, surveys conducted among farmers in Cameroon (Tindo et al. 2017), Benin (Houngbo et al. 2020), Botswana (Makale et al. 2022), Gabon (Cokola et al. 2023), and Uganda (Kalyebi et al. 2023) revealed that up to one‐third of respondents claimed to have observed the FAW between 2013 and 2015. However, the majority of the surveyed farmers reported observing the FAW from 2017 onward, suggesting that significant FAW damage only became apparent post‐2016 (Cokola et al. 2023; Kalyebi et al. 2023; Makale et al. 2022). This pattern reflects a typical lag phase in biological invasions, where introduced species remain at low, undetected levels before rapid population growth and noticeable impact occur (Durand et al. 2024). Hence, analyzing trends in adaptive evolution would reveal the traits responsible for the recent success of the FAW and aid in identifying potential targets for pest control (Wang et al. 2024; Yainna et al. 2022; Zhang, Li, et al. 2023).

While regional genomic studies have begun to characterize the invasion patterns of FAW across Asia (Rane et al. 2023), in‐depth country‐level population genomics analyses remain limited for several areas, including Malaysia. The pest was first detected in Peninsular Malaysia in February 2019 (IPPC 2019), and by January 2020, it had spread to all states (Jamil, Saranum, Mat, et al. 2021). Although FAW is currently limited to corn fields in Malaysia (Anonymous 2019), its known rice and maize host strains (*sfC* and *sfR*) raise concerns for cross‐host expansion. Malaysia's rice sector, which produced over 1.88 million metric tons of rice in 2019 (Dorairaj and Govender 2023; Department of Statistics Malaysia [DoSM] 2021) and is valued at RM 98.9 billion (O'Neill 2023; Statista 2023) could face elevated risk. Additionally, based on climate change patterns, Malaysia is projected to be most at risk of FAW outbreaks within Asia, alongside Northwest Myanmar, China, and Indonesia (Liu et al. 2020).

While recent large‐scale studies have included Southeast Asian samples (Rane et al. 2023; Wang et al. 2024; Zhang, Li, et al. 2023), this study contributes new whole‐genome data of Malaysian FAW individuals. Malaysia presents an agriculturally important context for studying local invasion dynamics, given its reliance on food imports and climatic suitability for FAW persistence. Our goal is to genetically characterize the Malaysian FAW, with the potential to offer insights into regional invasion pathways and evolutionary processes. Hence, our study sequenced, assembled, and examined the genomes of 42 novel Malaysian FAW samples to (i) explore FAW evolutionary trends across geographical regions, (ii) ascertain the origin and migration pathways of the Malaysian FAW, and (iii) uncover the genetic factors underlying the global success of FAW invasions. This study will be instrumental to the development of future pest management programs and policies.

## 2 Materials and Methods

### 2.1 Novel Samples for Population Genomic Analyses

**FIGURE 1:**
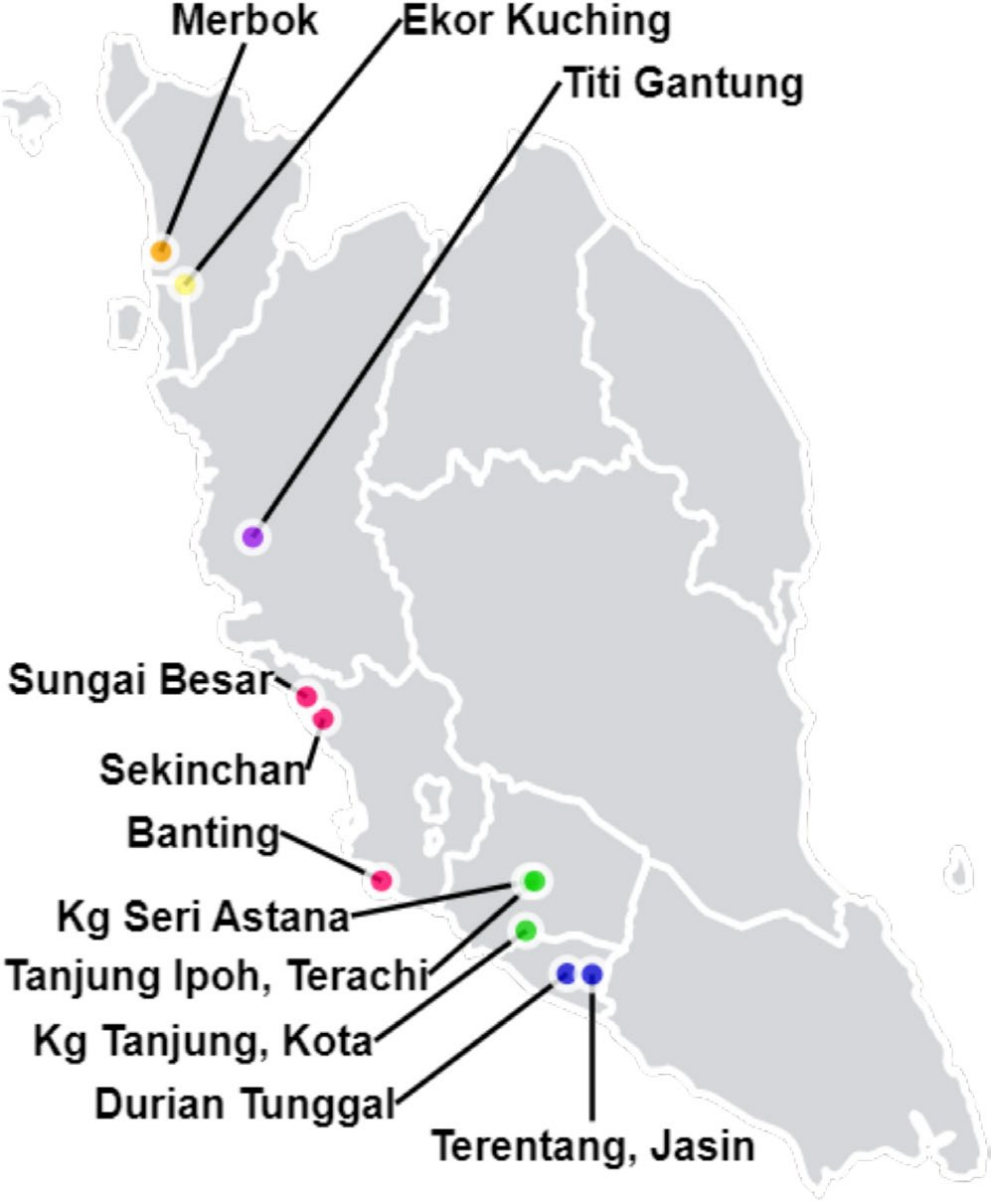
Geographical locations of field sampling sites in Peninsular Malaysia. Fall armyworm ( *S. frugiperda* ) samples for whole genome sequencing for population genomic analysis were collected from sweet corn fields located in Penang (yellow), Kedah (orange), Perak (purple), Selangor (red), Negeri Sembilan (green), and Melaka (blue).

FAW larvae were collected from corn fields in Penang, Kedah, Perak, Selangor, Melaka, and Negeri Sembilan (Figure 1 and Table S1). The samples were kept in large, ventilated plastic containers with soil and sweet corn until transportation to Monash University Malaysia. The FAW samples were then stored in absolute ethanol (99.9%), in sterile 15 mL Falcon tubes sealed with parafilm, at −20°C until DNA extraction.

### 2.2 DNA Extraction, Library Preparation, and Sequencing

Genomic DNA was extracted from whole, unsexed FAW larvae following a chemical‐based protocol (Sokolov 2000). Briefly, tissues were lysed in buffer containing Tris–HCl (50 mM), EDTA (10 mM), and SDS (1%) with Proteinase K, followed by RNase A treatment and potassium chloride precipitation. DNA was purified via chloroform extraction and isopropanol precipitation, washed twice with 80% ethanol, and resuspended in TE buffer. DNA quality and quantity were assessed using a NanoDrop spectrophotometer and agarose gel electrophoresis.

The extracted DNA samples were stored in TE buffer at −20°C until shipment for commercial sequencing. BGI Tech Solutions (Hong Kong) Co. Ltd. performed the library preparation using the BGI Optimal DNA Library Prep Kit. New genome sequence data were generated for the 42 FAW samples from Malaysia. This was performed by BGI using the DNBSEQ‐G400 platform, with 150 bp paired‐end read length.

### 2.3 Filtering, Mapping, and Variant Calling

**FIGURE 2:**
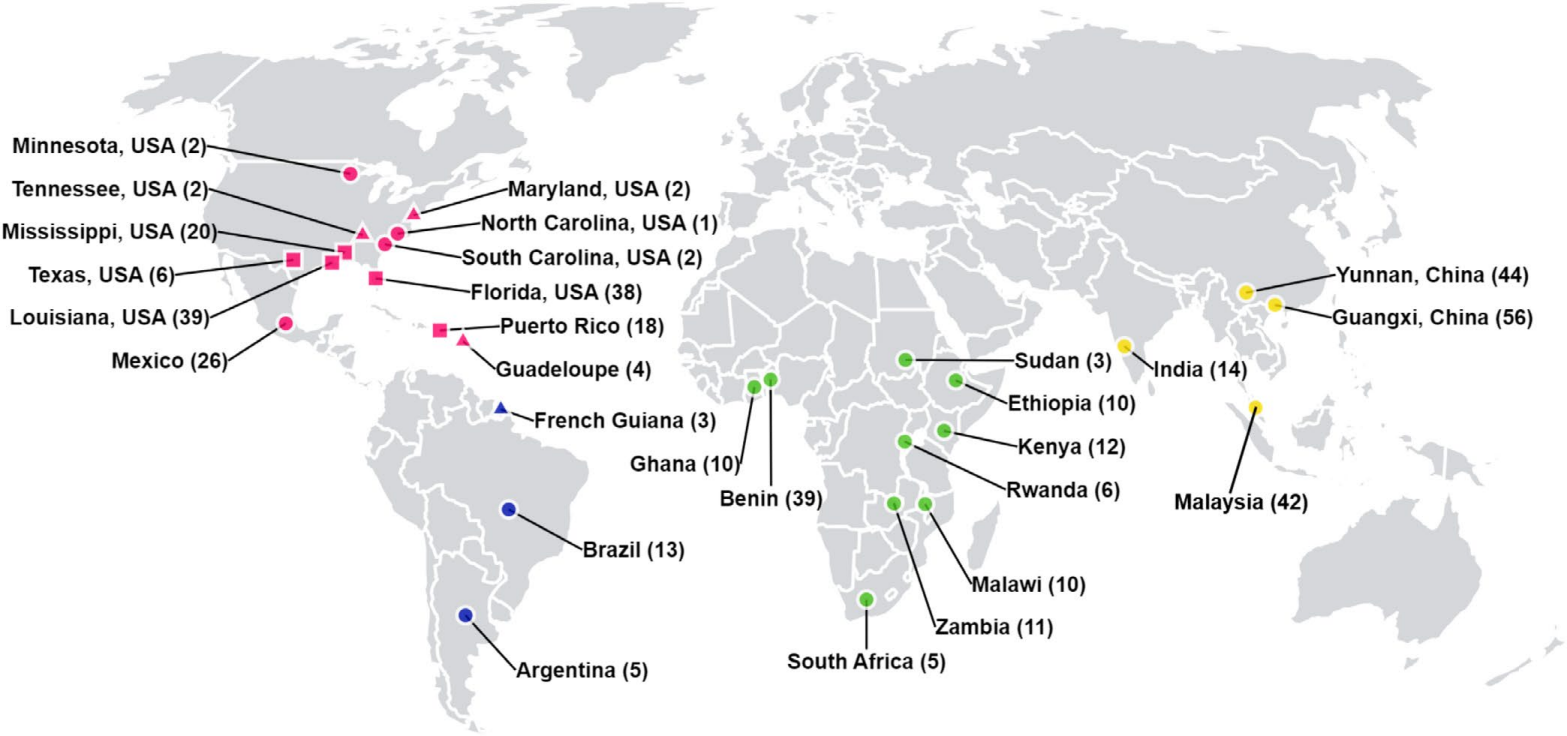
Geographical origins of the fall armyworm ( *S. frugiperda* ) samples surveyed for population genomic analyses. Samples obtained were from North America (red), South America (blue), Africa (green), and Asia (yellow). The number of samples collected from each location are listed in brackets. Each population contained either corn strain alone (circle), rice strain alone (triangle), or a mix of both corn and rice strain samples (square). Louisiana, USA, and Zambia samples were laboratory strains and not field samples.

In addition to the 42 newly generated whole‐genome sequences of Malaysian FAW samples (PRJNA1061840), a total of 401 different samples (179 from native populations and 222 from invasive populations) were obtained from previously published studies (Figure 2). These include samples from Benin, French Guiana, Guadeloupe, India, Mexico (PRJNA639295) (Yainna et al. 2022), Puerto Rico (PRJNA577869) (Gimenez et al. 2020), USA (PRJNA494340 and PRJNA639296) (Fiteni et al. 2022; Nam et al. 2020), Argentina, Brazil, Kenya, Puerto Rico, USA (PRJNA640063) (Schlum et al. 2021), Ghana, Malawi, Rwanda, Sudan, and Zambia (PRJNA591441) (Zhang, Li, et al. 2023), China, Ethiopia, Kenya, South Africa, and USA (CNP0001020) (Gui et al. 2022).

All analyses were performed using the Multi‐Modal Australian ScienceS Imaging and Visualization Environment (MASSIVE) high‐performance computing facility (Goscinski et al. 2014). Quality control of the new Malaysian FAW reads and the published resequencing reads was performed by BGI using *SOAPnuke* (Chen, Chen, et al. 2018) and *fastp* v0.22.0 (Chen, Zhou, et al. 2018), respectively. *FastQC* v0.12.1 (Andrews 2010) was used to perform quality control checks before and after read filtering (Ishizuka et al. 2023; Schlum et al. 2021; Tessnow et al. 2022).

Subsequently, all reads were aligned (Durand et al. 2022; Gimenez et al. 2020; Gui et al. 2022; Ishizuka et al. 2023; Rane et al. 2023; Schlum et al. 2021; Tay, Rane, Padovan, et al. 2022; Tessnow et al. 2022; Zhang et al. 2020) to a high‐quality reference genome (NCBI Assembly: GCA_023101765.3) (Tay, Rane, James, et al. 2022) using *bwa‐mem2* v2.2.1 (Vasimuddin et al. 2019). Mapping statistics were generated using *samtools* v1.9 (Danecek et al. 2021) and *Qualimap* v2.2.1 (García‐Alcalde et al. 2012). Duplicate, secondary, and unmapped reads were removed (Gimenez et al. 2020; Gui et al. 2022; Ishizuka et al. 2023; Nam et al. 2020; Rane et al. 2023; Tessnow et al. 2022; Zhang et al. 2020). Variant calling was performed using *bcftools* v1.9 (Danecek et al. 2021). The number of identified SNPs in 443 samples was 160,044,217. The variant calling format (VCF) file was then filtered using *VCFtools* v1.16 (Danecek et al. 2011). SNPs were retained based on the following parameters: biallelic SNPs only (Durand et al. 2022; Ishizuka et al. 2023), mapping quality score of ≥ 20 (Ishizuka et al. 2023), SNP genotype present in ≥ 90% of samples surveyed (Ishizuka et al. 2023; Zhang et al. 2020), Hardy–Weinberg equilibrium (HWE) exact test *p*‐value of ≥ 1e‐10 (Marees et al. 2018) and a minor allele frequency (MAF) of ≥ 0.01 (Ishizuka et al. 2023; Tay, Rane, Padovan, et al. 2022).

Five FAW samples (two from India, two from Florida, USA and one from Yunnan, China) were removed as they had more than 50% missing variant data (Schlum et al. 2021). SNPs were pruned for physical linkage and linkage disequilibrium (LD) (Gui et al. 2022; Rane et al. 2023; Tay, Rane, Padovan, et al. 2022) using *PLINK* v1.9 (Purcell et al. 2007). A window of 50 kb was set, with a window step size of 10 kb and a pairwise r^2^ correlation threshold of 0.1. The number of retained SNPs in 438 samples was 6,583,892.

### 2.4 Population Structure Analyses

Average weighted pairwise fixation index (*F*_ST_) and pairwise nucleotide diversity (π) were estimated with an untruncated window size of 100 kbp using *VCFtools* v1.16, alongside the inbreeding coefficient (*F*_IS_) (Danecek et al. 2011). Principal component analysis (PCA) was performed (Durand et al. 2022; Fiteni et al. 2022; Rane et al. 2023; Schlum et al. 2021; Tay, Rane, Padovan, et al. 2022; Yainna et al. 2022) using *PLINK* v1.9 (Purcell et al. 2007). Ancestry coefficient analysis was performed (Gimenez et al. 2020; Gui et al. 2022; Rane et al. 2023; Tay, Rane, Padovan, et al. 2022; Tessnow et al. 2022; Yainna et al. 2022) using *admixture* v1.3.0 (Alexander et al. 2009). The number of genetic clusters *K* was predefined from *K* = 2 to 15. The optimal *K* values were selected based on the lowest cross validation error. All output files from these analyses were exported to R version 4.2.1 (R Core Team 2022) and visualized using the *ggplot2* package (Wickham 2016).

### 2.5 Phylogenetic Analysis

A *TreeMix* input file format was created using the vcf2treemix.sh bash script (Meier 2018) and the plink2treemix.py python script (Pickrell and Pritchard 2012). *TreeMix* v1.13 (Pickrell and Pritchard 2012) was then run, with migration edges (*m*) set from *m* = 1 to 7 and 1000 bootstrap replications (Gui et al. 2022; Yainna et al. 2022). Output files were exported to R version 4.2.1 (R Core Team 2022) and visualized using the plotting_funcs. R Rscript (Pickrell and Pritchard 2012).

### 2.6 Genome Scan Detection of Potential Selective Sweeps

Potential targets of selective sweep were identified using *SweeD* v3.3.1 (Pavlidis et al. 2013), with a grid number per scaffold of 1000 (Durand et al. 2022; Fiteni et al. 2022; Gui et al. 2022; Yainna et al. 2022). Only the largest and localized scaffolds were assessed (Yainna et al. 2022). The 31 assessed scaffolds, which are comparable in number and scale to the FAW's full set of 31 chromosomes, accounted for 99.44% of the assembly (See www.ncbi.nlm.nih.gov/datasets/genome/GCF_023101765.2/). Outliers above the 99.9th percentile of the composite likelihood ratio (CLR) test were considered as loci under selective sweep. SNPs under selective sweep were identified (Ishizuka et al. 2023) using the R package *pcadapt* (Privé et al. 2020). Outliers of population differentiation were identified with a false discovery rate of 0.01. Only outlier SNPs identified by *pcadapt* that fell within the selected *SweeD* regions were retained as candidate markers under selection. The associated genes under selective sweep were then extracted from the published annotation of the chosen FAW reference genome, using *BEDTools* v2.31.0 (Quinlan and Hall 2010).

## 3 Results

### 3.1 Genome Mapping Statistics

High‐quality, paired‐end short‐read sequencing data were generated from a total of 42 novel Malaysian FAW samples, achieving an average genome coverage of 32.09X (SE = ±1.70) (Table S2). Sequencing reads were virtually free of error, with 90.61% ± 0.64% of reads possessing a quality score of ≥ 30. On average, 97.07% ± 0.14% of the Malaysian FAW sample reads aligned to the reference genome, indicating high‐quality alignment of the sample reads (Malinsky et al. 2016). The GC content of the novel Malaysian FAW genomes was 36.71% ± 0.04%, similar to previous reports (Gimenez et al. 2020; Gouin et al. 2017; Gui et al. 2022; Tay, Rane, James, et al. 2022; Xiao et al. 2020; Zhang et al. 2020). The mean mapping quality of all Malaysian samples was 49.81 ± 0.06, indicating a mapping accuracy of almost 99.999%. For the resequencing samples, an average of 93.28% ± 0.41% reads aligned to the reference genome, with 77.41% ± 0.49% properly paired and only 2.38% ± 0.04% as singletons.

### 3.2 Population Structure

**FIGURE 3:**
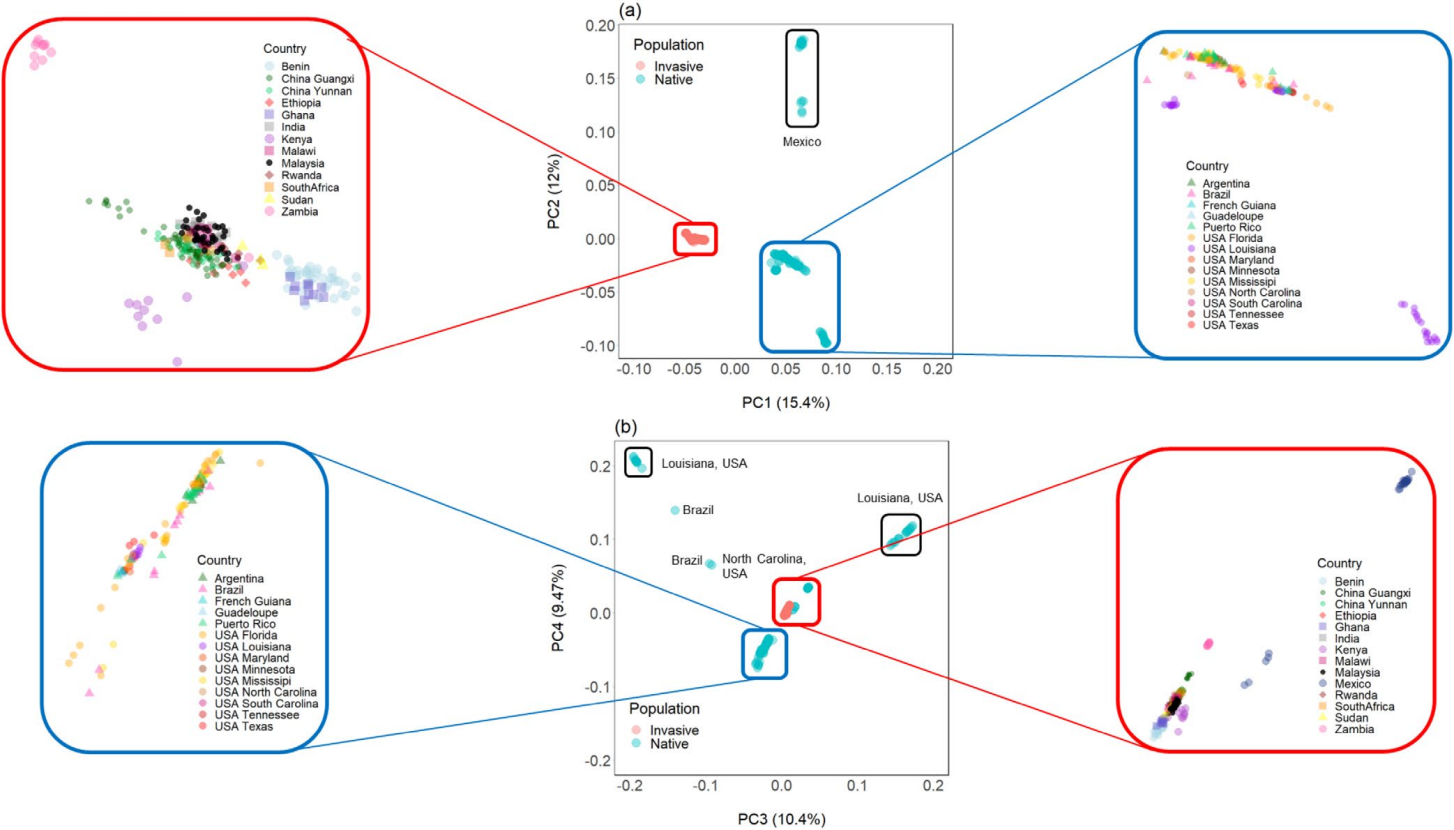
Principal component analysis of fall armyworm ( *S. frugiperda* ) samples using 6,583,892 SNPs. (a) all populations plotted along PC1 and PC2; (b) all populations plotted along PC3 and PC4. Numbers in parentheses represent the percentage of genetic variation explained by each principal component (PC).

In the PCA, 20.37% of the total variance was explained by the first 20 PCs (Figure S1). Native and invasive populations clearly separated from each other along PC1 (Figure 3a). Within this invasive cluster, some population‐level structure was visible: Kenya, Zambia, and a subset of China (Guangxi) showed mild separation from the central mixed group that included individuals from Malaysia, India, Malawi, Sudan, Rwanda, Ethiopia, and South Africa. Most notably, Benin and Ghana did *not* overlap with the main mixed cluster of invasive populations. Along PC2, Mexico separated away from all other native populations (Figure 3a). Louisiana, USA samples formed three distinct clusters along all PCs.

**FIGURE 4:**
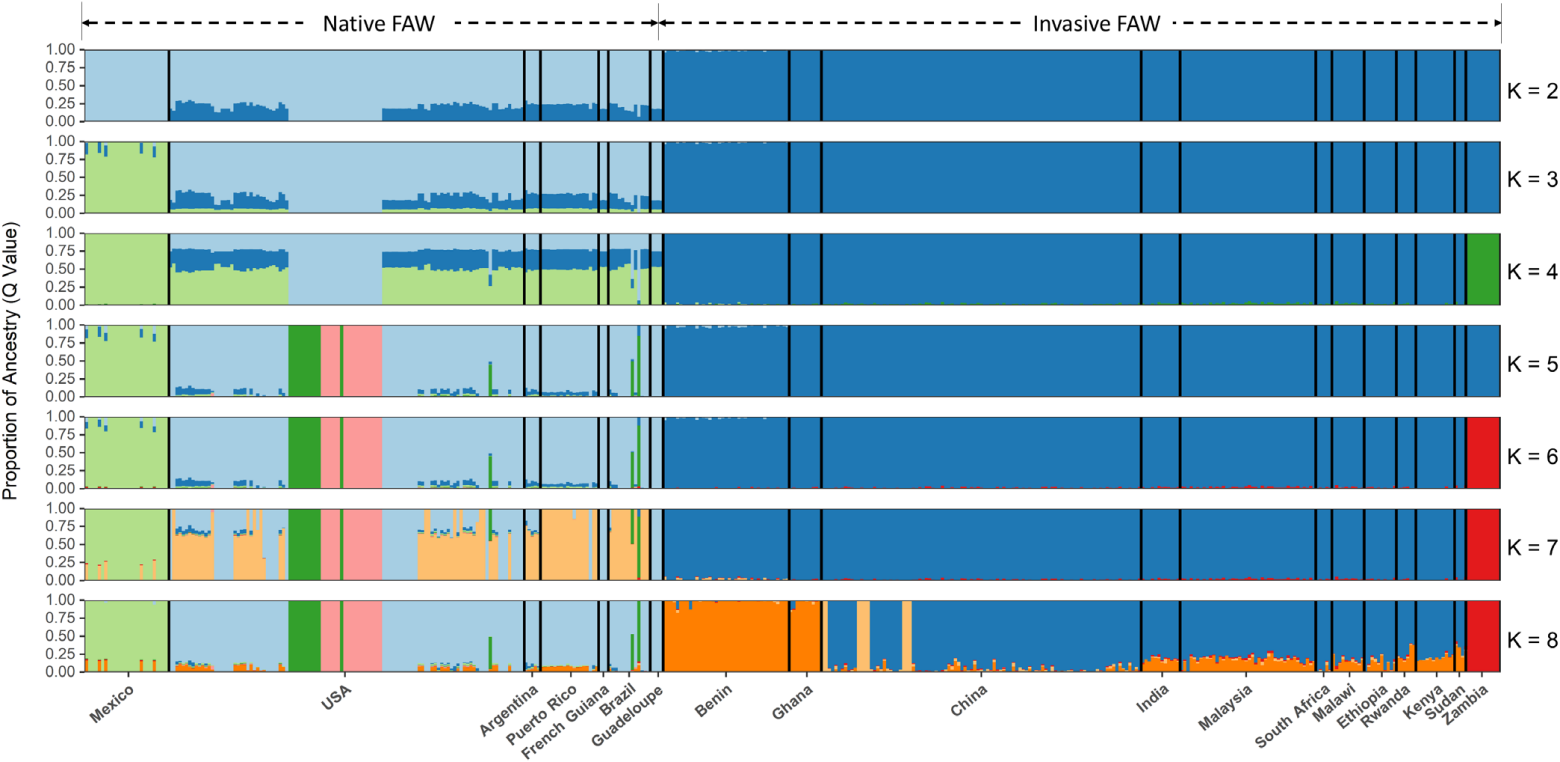
Admixture analysis of fall armyworm (*S. frugiperda*) samples by geographical location using 6,583,892 SNPs. The lowest cross‐validation (CV) error values were obtained at *K* = 6 and 8. *K* = 9 and onward was excluded as no new information could be determined from the admixture plots, and an increasing trend in CV error values was observed. *K: number of ancestral populations*.

The admixture results support the findings observed in the PCA (Figure 3). The clustering pattern from *K* = 2 onward revealed that invasive FAW populations shared a single common ancestor, distinct from native populations (Figure 4). The lowest CV error values were obtained at *K*= 6 and 8 (Figure S2). At *K* = 6, the majority of invasive populations clustered together (dark‐blue), with the exception of Zambia (red). Among the native populations, four clusters are apparent, with Mexico (light‐green) and Louisiana, USA (dark‐green and pink) separating away from all other native populations, including a few other samples from Louisiana, USA (light‐blue). At *K* = 8, Benin and Ghana (orange) form another cluster, away from the other invasive populations (dark‐blue). From *K* = 9 onward, no new information can be determined from the admixture plots, and an increasing trend in CV error values is observed (Figure S2).

### 3.3 Population Statistics and Gene Flow

**FIGURE 5:**
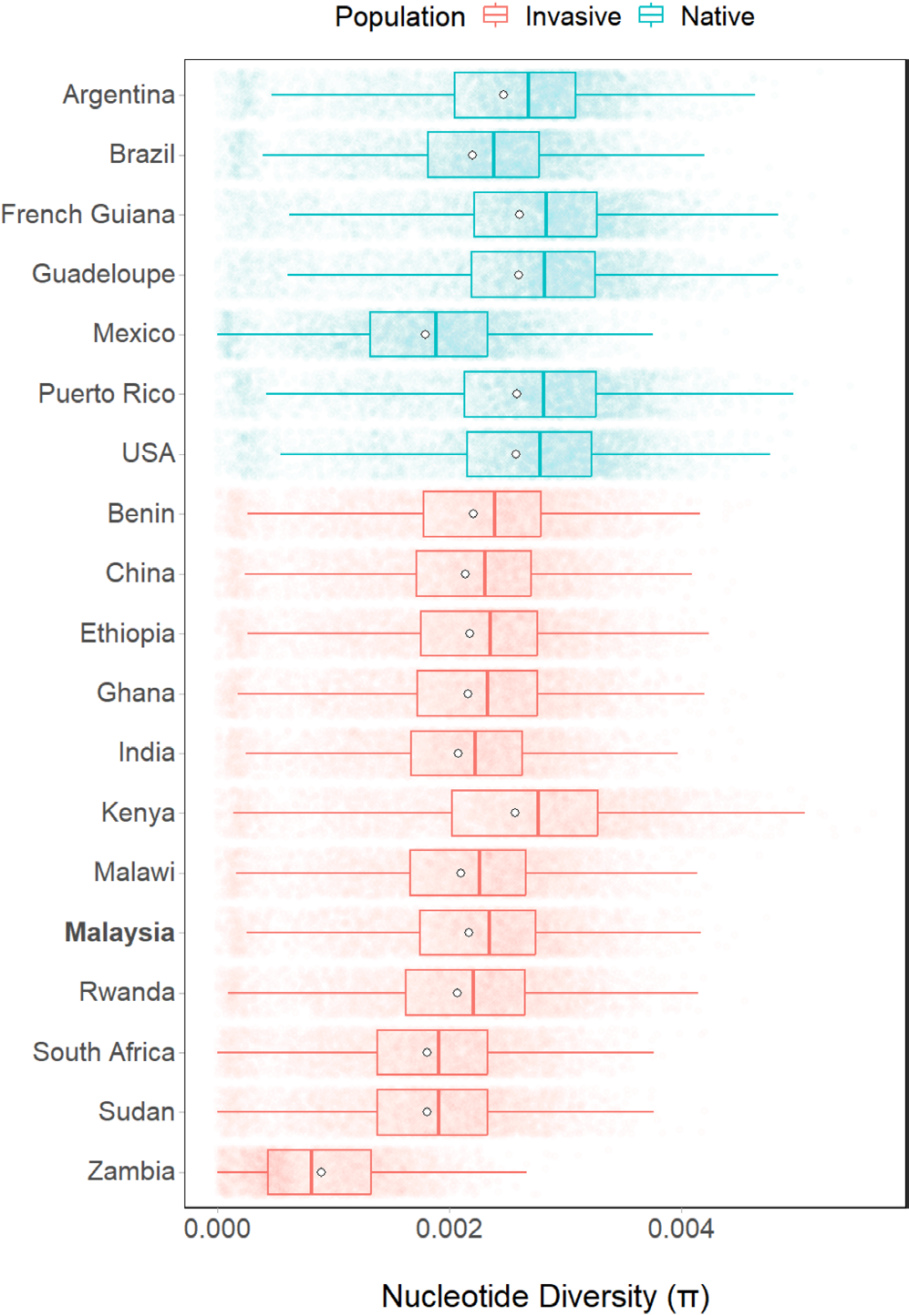
Nucleotide diversity of fall armyworm ( *S. frugiperda* ) samples by geographical region. The boxes represent the interquartile range, the line in between represents the median, the whiskers represent the values outside the interquartile range and the white circles represent the mean.

In this study, π mostly varied across a narrow range of 10^−3^ (Figure 5), similar to earlier studies (Gui et al. 2022; Nagoshi et al. 2021; Schlum et al. 2021). As expected, native populations ($\bar{\pi }$ = 2.55 ± 0.02 × 10^−3^) were significantly more polymorphic than invasive populations ($\bar{\pi }$ = 2.18 ± 0.01 × 10^−3^) on average (*t*(7636) = 17.971, *p*‐value < 0.001).

**FIGURE 6:**
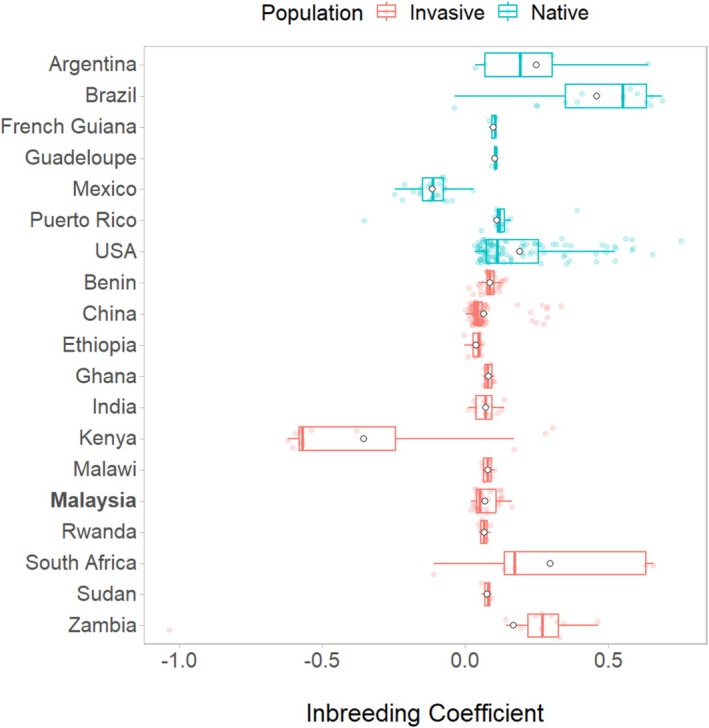
Inbreeding coefficient of fall armyworm ( *S. frugiperda* ) samples by geographical region. The boxes represent the interquartile range, the line in between represents the median, the whiskers represent the values outside the interquartile range and the white circles represent the mean.

On the other hand, *F*_is_ varied across a much broader range (Figure 6), with native populations (*F*_is_ = 0.16 ± 0.01) being significantly more inbred than invasive populations (*F*_is_ = 0.6 ± 0.01) on average (*t*(333) = −5.217, *p*‐value < 0.001). Moreover, Zambia exhibited the lowest nucleotide diversity (π = 0.9 ± 0.01 × 10^−3^) and one of the highest inbreeding coefficients (*F*_is_ = 0.168), suggesting the presence of highly related individuals. This likely explains Zambia's high *F*_ST_ values and extreme drift observed in downstream analyses.

**FIGURE 7:**
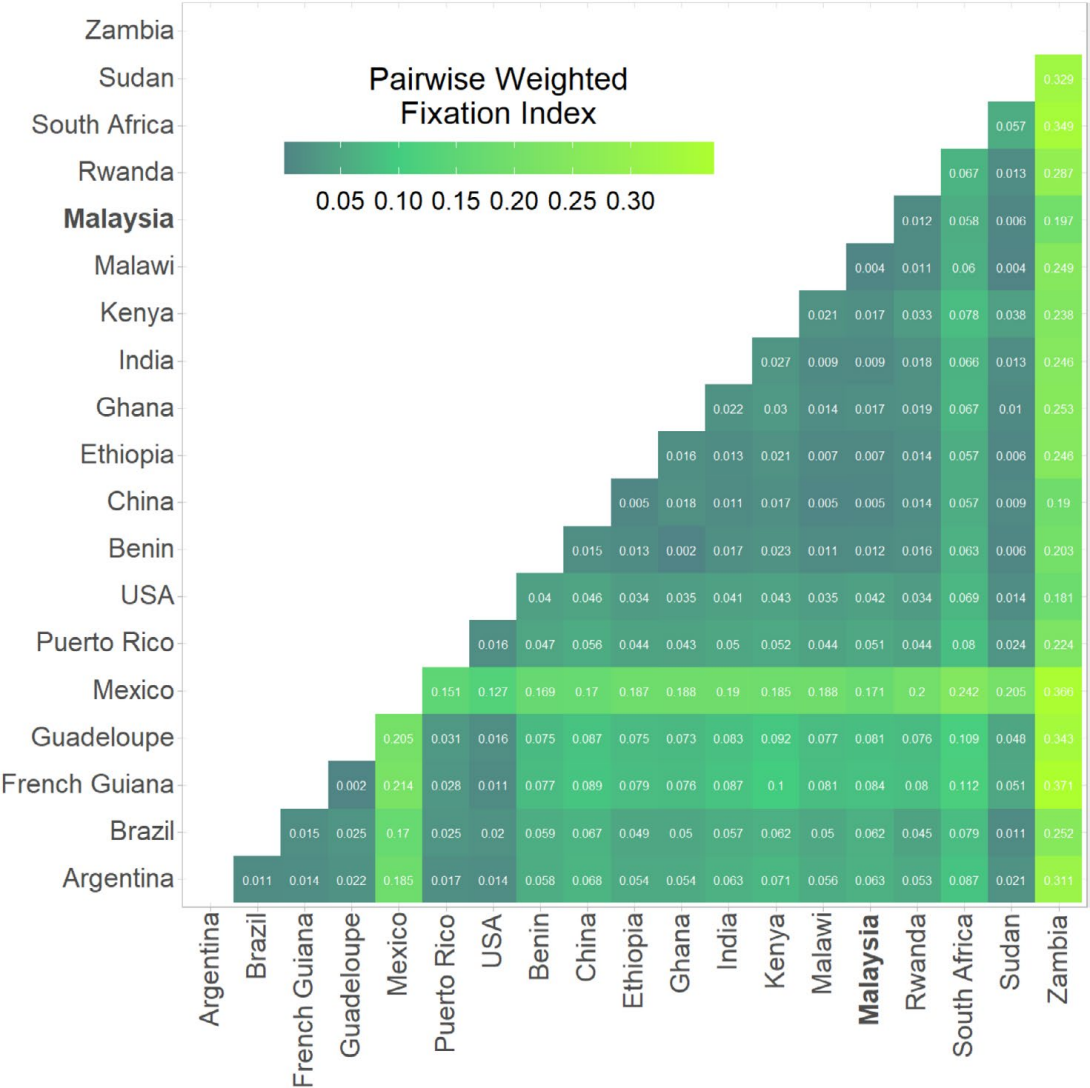
Heatmap representing pairwise weighted fixation indices (*F*_ST_) of fall armyworm ( *S. frugiperda* ) samples by geographical region. Low values/darker colors indicate ongoing gene flow, whereas high values/light colors indicate limited gene flow between populations.

The *F*_ST_ values also indicated strong population differentiation between invasive and native populations (Figure 7). Zambia was the most genetically distinct population with the highest *F*_ST_ values, followed by Mexico. Ongoing gene flow was evident between Malaysian and other invasive populations, namely Malawi, followed by China, Sudan, Ethiopia, and India (*F*_ST_ ≤ 0.009).

### 3.4 Phylogenetic Analysis

**FIGURE 8:**
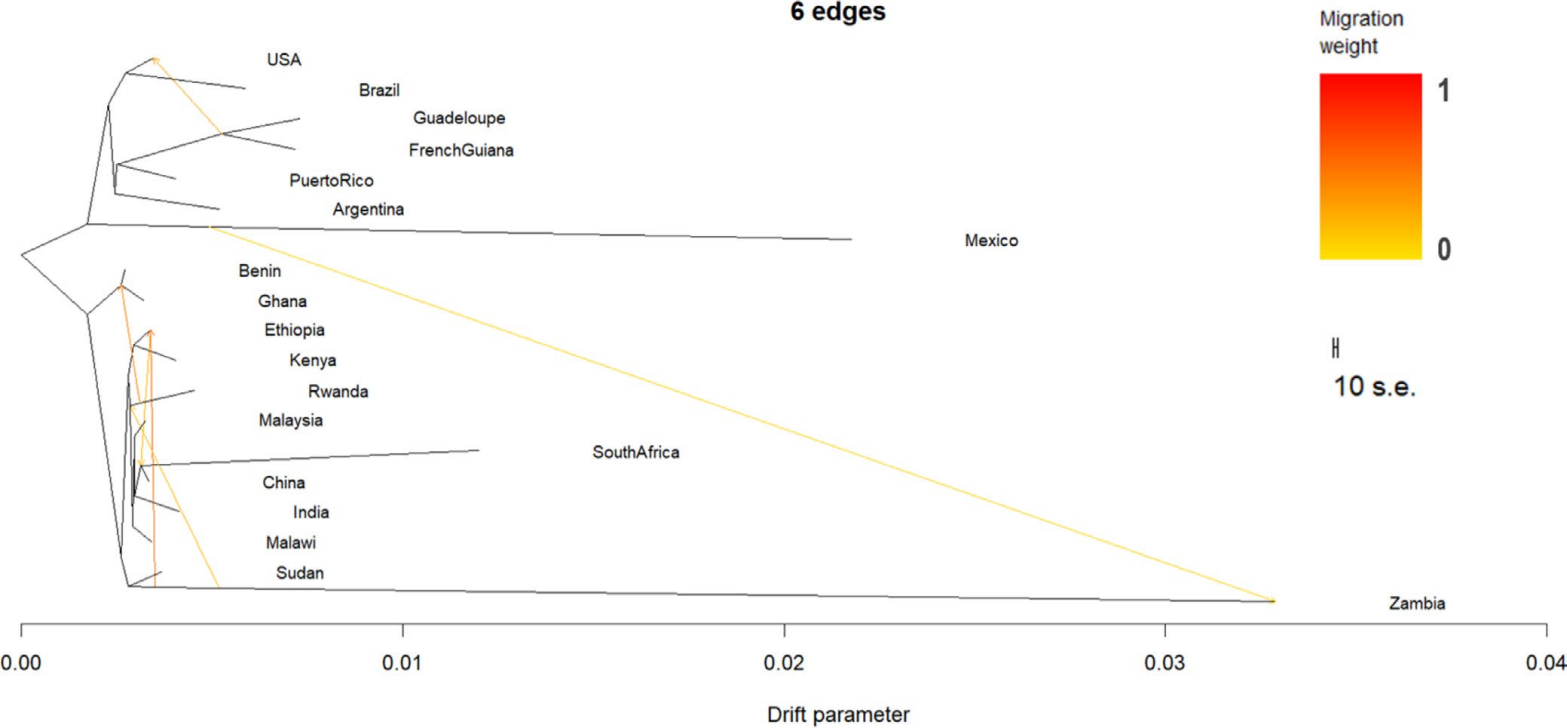
Fall armyworm ( *S. frugiperda* ) tree topology using 6,583,892 SNPs, with six migration events inferred by TreeMix. The arrows indicate gene flow between populations, with arrowheads representing the direction and color representing the weight of the migration event. Migration events from Zambia to Ethiopia (52.20%), Rwanda to Ghana and Benin (43.63), French Guiana to the USA (30.07%), Ethiopia to South Africa and China (20.18%), Zambia to Rwanda, South Africa, China, Malaysia, and India (19.53%), and Mexico to Zambia (7.78%) were observed.

Based on residual analysis (Figures S3, S4), the model that best fit the data in the *TreeMix* phylogenetic analysis contained six migration events (Figure 8). Native populations split away from all invasive populations. Zambia, followed by Mexico, possessed the highest drift parameter values, in concordance with their high *F*_ST_ values, including a notably high Zambia–Mexico value (*F*_ST_ = 0.366; Figure 7). This makes the TreeMix‐inferred migration edge between these two populations highly unexpected and likely artifactual, driven by high drift in both. Similarly, inferred gene flow from Zambia to other invasive populations likely reflects methodological artifacts stemming from Zambia's high drift and low diversity, rather than genuine directional gene flow. Among the invasive populations, Benin and Ghana separated away from the majority of the invasive populations, whereas Malaysia formed a monophyletic clade with India, China, and South Africa.

### 3.5 Genome‐Wide Selection Scan

**FIGURE 9:**
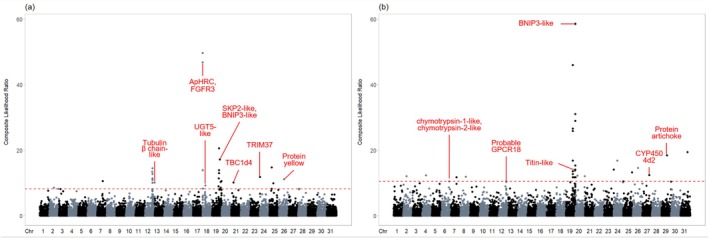
Loci under positive selection in invasive fall armyworm ( *S. frugiperda* ) populations as determined by SweeD. The y‐axis represents the composite likelihood of being targeted by selective sweeps whereas the x‐axis represents the chromosomes. Loci above the dashed red lines are among the top 0.1% regions being selected for. (a) loci under selection specific to all invasive populations; (b) loci under selection specific to the Malaysian population alone. *ApHRC: Histidine‐rich calcium‐binding protein‐like; FGFR3: Fibroblast growth factor 3; UGT5‐like: UDP‐glycosyltransferase UGT5‐like; SKP2‐like: S‐phase kinase‐associated protein 2‐like; BNIP3: BCL2/adenovirus E1B 19 kDa protein‐interacting protein 3‐like; TRIM37: E3 ubiquitin‐protein ligase TRIM37; TBC1d4: TBC1 domain family member 4; GPCR18: G‐protein coupled receptor 18; CCS‐like: Copper chaperone for superoxide dismutase‐like; CYP450 4d2: Cytochrome P450 4d2*.

Using the top 0.1% composite likelihood ratios, 31 regions in both all invasive FAW populations and the Malaysian FAW population alone were identified as undergoing selective sweep, with most being found on chromosome 19 (Figure 9). Of the selected regions, 16 and 26 contained highly differentiated, outlier SNPs which were used to identify the exact genes undergoing adaptive evolution in all invasive FAW populations and the Malaysian FAW population, respectively. In all invasive FAW populations, 72 genes were identified, with 46 genes encoding characterized proteins, whereas in the Malaysian FAW population, 55 genes were identified with 37 encoding characterized proteins (Table S3). Selection scan of native FAW populations shows little overlap with the invasive sweep profiles, indicating distinct adaptive pressures acting on invasive populations (Figure S5).

## 4 Discussion

As a globally significant pest, the FAW has attracted significant interest from researchers aiming to unravel its origins and ongoing evolutionary processes. Most existing studies have focused on FAW populations from the Americas, Africa, and Asia; fewer studies have focused in detail on local Malaysian populations using high‐resolution whole‐genome data. Our study builds on these efforts by presenting 42 newly sequenced Malaysian FAW genomes, analyzed in the context of 396 global genomes from 18 countries. Our analysis focuses on Malaysia as a case study to examine localized FAW invasion dynamics. By doing so, we provide additional insight into how regional trade, natural migration, and historical introductions have shaped genetic patterns in Malaysia, while contributing new comparative evidence to the growing body of literature on FAW spread across Asia. Our results suggest that multiple introduction pathways, ongoing gene flow, and adaptive evolution have jointly influenced the genetic structure of Malaysian FAW populations.

### 4.1 Geographical Evolutionary Patterns

Consistent with previous research (Gui et al. 2022; Wang et al. 2024; Yainna et al. 2022; Zhang, Li, et al. 2023), the present study showed distinct population differences between invasive and native FAW samples, likely due to the founder effect (Provine 2004) and varying selection pressures. Invasive populations also displayed lower genetic polymorphism than native populations (Figure 5), supporting the view that native FAW populations are ancestral.

Contrary to the hypothesis that West Africa served as the primary source for FAW invasions into Asia and East Africa, following the official incidence reporting timeline (Cock et al. 2017; Goergen et al. 2016), our data does not support this model. If Ghana and Benin were the founding populations, we would expect to observe admixed individuals within the central invasive PCA cluster; however, such patterns are absent (Figure 3). Additionally, these populations exhibit distinct ancestral components, suggesting minimal genetic contribution to the invasive populations in Asia and East Africa (Figure 4). TreeMix analyses further indicate that migration events involving Benin and Ghana are more likely to be incoming rather than outgoing (Figure S4).

Instead, our findings align with the growing body of evidence supporting multiple introductions of FAW into Asia (Nagoshi et al. 2022; Rane et al. 2023; Tay, Rane, Padovan, et al. 2022). The Malaysian FAW populations form a monophyletic clade with those from India, China, and South Africa, suggesting shared ancestry (Figure 8). While previous studies have reported admixture between native and invasive populations, our data suggest that such mixing may be less extensive, potentially due to earlier separation events prior to 2016, with hybridization occurring predominantly within the Eastern Hemisphere (Wang et al. 2024). Moreover, strong gene flow between Malaysia and Malawi indicates potential connectivity between Southeast Asian and East African populations (Figure 7), and possible east‐to‐west movement of FAW (Rane et al. 2023). Such findings underscore the complexity of FAW invasion dynamics and the need for continuous monitoring and comprehensive genomic analyses to unravel these patterns.

### 4.2 Origins and Possible Introduction Pathways of FAW Into Malaysia

Our results suggest that invasive FAW was introduced via multiple pathways. The Malaysian FAW samples were particularly closely related to those from India (Figure 3; Figure 8) and displayed strong and ongoing gene flow with the populations in China (Figure 7; Figure 8). As Malaysia relies heavily on agricultural imports, with 60% of its food sourced externally (Editor, 2022), particularly from China (11.6%) and India (8.1%) (DoSM 2021), these trade routes likely facilitated the introduction of FAW. These countries were also the top exporters of cut flowers to Malaysia in 2019 (World Integrated Trade Solution 2025) which have been previously linked to FAW dispersal (Tay, Rane, Padovan, et al. 2022), further supporting this hypothesis.

Natural migration may also have contributed to FAW dispersal into Malaysia. Models predicting multigenerational migrations of FAW across East and Southeast Asia are driven by monsoon winds that favor southward migration from China toward the Indochina Peninsula, September onward (Li et al. 2020; Wu, Jiang, et al. 2021; Zhang, Huang, et al. 2023). This predicted migration corridor is similar to the migration route of other moth pests (Feng et al. 2008; Feng et al. 2003; Feng et al. 2005). Previous studies have provided genetic evidence for such migration patterns (Xu et al. 2016). However, model‐based migration trajectories are influenced by detection timing and may not accurately reflect actual source–sink relationships, as shown by discrepancies in FAW introductions to Australia (Qi et al. 2021; Rane et al. 2023).

An intriguing finding is the genetic similarity between Malaysian and Malawian FAW populations, as indicated by limited population differentiation (Figure 3; Figure 6). Previous studies have also found that Southeast Asian and East African FAW populations have similar genetic ancestry, despite their geographical distance (Tay, Rane, Padovan, et al. 2022). Malawi has limited agricultural trade ties with Asia, with 99.9% of its corn exports being allotted to other African countries. Alternatively, natural migration appears to be an unlikely introduction route, as it would require movement across thousands of kilometers across the Indian Ocean. However, the dragonfly *Pantala flavescens* is known for its transoceanic migrations every spring from East Africa to India, covering nearly 18,000 km (Ranjan et al. 2023). Whether the FAW is capable of completing such a flight is yet to be elucidated.

To better understand the origins of the Malaysian FAW, it is advisable to analyze FAW samples from across Southeast Asia. Indonesia in particular is a strong candidate, being the top agricultural importer for Malaysia in 2020 (DoSM 2021). Moreover, the island of Sumatra, where the FAW was first officially reported in Indonesia (Sartiami et al. 2020), is southwest of Malaysia. Thus, the aforementioned monsoon patterns would favor FAW migration from Indonesia to Malaysia in spring. Alternatively, FAW samples from Thailand are of great interest, being among the top four agricultural importers for Malaysia (DoSM 2021), and because the Malaysian FAW was first detected in the Thailand‐bordering state of Perlis (International Plant Protection Convention 2019). Inclusion of additional FAW samples from the Indo‐China Peninsula could help confirm the relationships and migratory patterns of the East and Southeast Asian FAW.

### 4.3 Factors Contributing to the Recent Success of the Malaysian FAW

A variety of genes were targeted by selective sweep specific to all invasive FAW populations, including histidine‐rich calcium‐binding protein‐like, which is involved in plant defense response (Wang et al. 2020), as well as protein yellow (Drapeau et al. 2006) and tubulin beta chain‐like (Li et al. 2022), which may contribute to the FAW's large reproductive capacity. For the Malaysian FAW, notable outlier SNPs were present in genes encoding protein artichoke, which is crucial for sensory ciliated sensilla function (Andrés et al. 2014), titin‐like, essential for insect flight muscles contraction (Yuan et al. 2015) and probable G‐protein coupled receptor No18, involved in salivary gland function (Gold et al. 2020; Soohoo‐Hui et al. 2021). While the functional roles of some of these genes remain unvalidated in FAW, together, they may enhance the pest's ability to exploit local resources and adapt to the new environment.

In addition to these adaptive traits, our findings indicate the importance of insecticide resistance in FAW's global success. In invasive populations, insecticide defense response genes including TBC1d4 against chlorantraniliprole (Meng et al. 2023) and UGT5‐like against pyrethroids (Xu et al. 2020), as well as CYP4504d2 against spinetoram (Gao et al. 2022) and coumarin (Xia et al. 2023) in the Malaysian FAW were identified as candidates for positive selection. While these findings are consistent with reports from Malaysian farmers noting reduced insecticide efficacy (Jamil, Saranum, Saleh Hudin, and Anuar Wan Ali 2021), phenotypic assays are still required for confirmation. Alternatively, our identification of chymotrypsin, targeted by selective sweep, suggests that resistant corn varieties inhibiting this digestive enzyme could be an effective pest control strategy (Kim et al. 2022).

Climate change may have also contributed to the recent increase in FAW outbreaks. Ocean warming is expanding the earth's tropic belt (Yang et al. 2020), creating more habitats with optimal temperatures for FAW survival. Maximum entropy species distribution models indicate that FAW prevalence in Asia and Africa will further expand in the future due to increased habitat suitability (Ramasamy et al. 2022; Zacarias 2020). On a related note, among loci targeted by selective sweep in invasive populations, we identified two ubiquitination‐related (S‐phase kinase‐associated protein 2‐like, E3 ubiquitin‐protein ligase TRIM37) and two apoptosis‐related (BCL2/adenovirus E1B 19 kDa protein‐interacting protein 3, fibroblast growth factor 3) candidate genes under selection that could potentially be involved in the biological processes behind FAW heat tolerance (Vatanparast and Park 2022). These genomic signals provide starting points for further investigation and underscore the need for urgent control measures, as climate change could facilitate the pest's spread to even more regions.

### 4.4 Implications for FAW Management in Malaysia and Southeast Asia

To enhance regional pest management, it may be beneficial for the Malaysian authorities to consider initiating the usage of the FAW Monitoring and Early Warning System (FAMEWS) app among local farmers to facilitate communication of FAW migratory movement surveillance data and to provide educational resources (FAO 2020). Furthermore, cross‐border collaboration, particularly through initiatives like the ASEAN FAW working group under the Asia‐Pacific Plant Protection Commission (APPPC; see https://www.aseanfawaction.org/), will be essential for coordinating efforts across the region and exchanging best practices for pest management.

## 5 Conclusion

This study represents an advancement in our understanding of the global spread and adaptation of the FAW. By generating and analysing 42 novel Malaysian FAW genomes in conjunction with global populations, we find evidence that supports the theory of multiple independent introductions into Asia, with Malaysian FAW populations showing strong genetic affinity to those from India, China, and African nations. We find no genomic support for Ghana or Benin serving as source populations for the broader Asian or East African FAW invasions. Instead, these populations appear genetically distinct, likely reflecting complex introduction routes and region‐specific adaptation histories. The findings also highlight how trade, monsoon‐driven migration, and possible long‐distance dispersal may have shaped FAW presence in Malaysia. While some evidence of admixture exists, our data suggest that mixing between native and invasive populations may be less widespread than previously reported, with differentiation patterns indicating early divergence and localized evolution. Genome‐wide scans suggest that recent outbreaks are linked to adaptive evolution, with key genetic changes conferring resistance to insecticides and heat, traits that have fueled the pest's rapid global success. This work not only highlights the urgent need for immediate and targeted action to combat FAW but also provides a blueprint for understanding other invasive species through the lens of population genomics. Ultimately, our results add to a growing body of evidence supporting the role of multiple introductions and adaptive evolution in shaping one of the world's most invasive pest species, informing more localized and genetically informed pest management strategies.

## Consent

All authors approved and consented to the publication.

## Conflicts of Interest

The authors declare no conflicts of interest.

## Supporting information


Appendix S1.

## Data Availability

The raw reads sequencing data is available in FASTQ format at NCBI Sequence Read Archive (SRA) repository with the BioProject ID PRJNA1061840.
