# Supplementary material for: From the Americas to Southeast Asia: Navigating The Genomic Waves of Fall Armyworm (Spodoptera frugiperda) Invasions
Source: Evol Appl. 2025 Jul 31;18(8):e70139. doi: 10.1111/eva.70139 (PMC12312211; doi:10.1111/eva.70139)
Supplement: Supplementary file 1 — Appendix S1. [file EVA-18-e70139-s001.docx]

## APPENDIX

**Table S1.** **Samples used for the population genomic analyses.** The population code, location, geographical coordinates, and number of samples of the Malaysian fall armyworm (*S. frugiperda*) used in whole genome sequencing for population genomic analysis. *n*: number of samples.

| **Date of Collection** | **State** | **Code** | **Location** | **GPS Coordinates** | ***n*** |
| --- | --- | --- | --- | --- | --- |
| 25^th^ November 2019 | Penang | PN | Mukim 3, Ekor Kuching | 5.55, 100.52 | 3 |
| 28^th^ November 2019 | Kedah | KD | Kg Pondok, Merbok | 5.70, 100.41 | 3 |
| 5^th^ December 2019 | Perak | PR | Titi Gantung, 32600 | 4.36, 100.84 | 4 |
| 13^th^ June 2023 | Selangor | SK | Sekinchan | 3.51, 101.18 | 4 |
|  |  | SG | Sungai Besar | 3.61, 101.10 | 4 |
|  |  | BT | Kg Jalan Tali air Banting | 2.74, 101.45 | 4 |
| 14^th^ June 2023 | Malacca | TG | Durian Tunggal | 2.31, 102.33 | 4 |
|  |  | JS | Terentang, Jasin | 2.30, 102.45 | 4 |
| 15^th^ June 2023 | Negeri Sembilan | AT | Kg Seri Astana | 2.74, 102.15 | 4 |
|  |  | TI | Tanjung Ipoh, Terachi | 2.74, 102.180 | 4 |
|  |  | TK | Kg Tanjung, Kota | 2.51, 102.13 | 4 |
|  |  | **Total** |  |  | **42** |

**Table S2. Mapping Statistics of 42 Novel Malaysian Fall Armyworm (*Spodoptera frugiperda)* Samples.**

| ***State*** | ***Sample*** | **Clean Reads** | **Q30** | **Mapping Rate** | **Properly Paired** | **Singletons** | **Mean Coverage** | **GC content** | **Mean Mapping Quality** | **No. of Variants** | **het/hom ratio** |
| --- | --- | --- | --- | --- | --- | --- | --- | --- | --- | --- | --- |
| **Penang** | **PN01** | 20,002,938 | 94.80% | 98.38% | 87.35% | 0.87% | 13.29 | 36.88% | 50.59 | 6499761 | 0.114 |
|  | **PN03** | 20,124,204 | 89.69% | 97.11% | 79.40% | 1.97% | 13.17 | 37.15% | 50.21 | 6498604 | 0.113 |
|  | **PN04** | 20,082,283 | 90.25% | 97.32% | 80.28% | 1.88% | 13.17 | 36.85% | 50.35 | 6494628 | 0.113 |
| **Perak** | **PR01** | 20,020,323 | 91.84% | 95.64% | 80.71% | 1.48% | 12.76 | 37.42% | 50.24 | 6497465 | 0.114 |
|  | **PR02** | 20,069,699 | 91.90% | 97.76% | 82.67% | 1.51% | 13.20 | 37.09% | 50.45 | 6498603 | 0.113 |
|  | **PR03** | 20,155,781 | 90.36% | 97.12% | 80.41% | 1.79% | 13.22 | 37.13% | 50.33 | 6500186 | 0.113 |
|  | **PR04** | 20,084,490 | 94.52% | 97.51% | 86.49% | 0.93% | 13.14 | 36.41% | 50.48 | 6502706 | 0.118 |
| **Kedah** | **KD01** | 18,873,463 | 93.38% | 98.13% | 84.86% | 1.14% | 12.46 | 36.46% | 50.52 | 6494888 | 0.114 |
|  | **KD02** | 20,079,343 | 92.20% | 98.55% | 88.18% | 0.74% | 13.21 | 36.00% | 50.60 | 6475025 | 0.108 |
|  | **KD04** | 20,097,461 | 94.77% | 97.17% | 86.27% | 1.01% | 12.80 | 36.36% | 50.26 | 6497738 | 0.120 |
| **Selangor** | **SK01** | 60,202,656 | 84.04% | 96.60% | 80.33% | 2.15% | 37.86 | 36.88% | 49.50 | 6545204 | 0.140 |
|  | **SK02** | 60,195,997 | 84.03% | 96.73% | 80.61% | 2.13% | 37.72 | 36.84% | 49.35 | 6541134 | 0.138 |
|  | **SK04** | 60,187,308 | 83.63% | 96.63% | 80.34% | 2.16% | 37.86 | 36.90% | 49.39 | 6540348 | 0.135 |
|  | **SK05** | 60,255,904 | 83.55% | 96.68% | 79.59% | 2.25% | 37.88 | 36.74% | 49.38 | 6541150 | 0.139 |
|  | **SG01** | 60,157,748 | 83.55% | 96.80% | 80.86% | 2.17% | 37.66 | 36.86% | 49.39 | 6544848 | 0.140 |
|  | **SG02** | 60,176,704 | 82.86% | 96.62% | 78.84% | 2.43% | 37.85 | 36.84% | 49.35 | 6538366 | 0.138 |
|  | **SG05** | 60,146,456 | 83.98% | 96.74% | 79.52% | 2.30% | 37.88 | 36.40% | 49.36 | 6543199 | 0.139 |
|  | **SG06** | 60,054,880 | 84.06% | 96.88% | 79.84% | 2.22% | 37.88 | 36.79% | 49.33 | 6543946 | 0.142 |
|  | **BT01** | 60,106,669 | 88.89% | 97.17% | 80.79% | 1.95% | 38.61 | 36.69% | 49.67 | 6541975 | 0.139 |
|  | **BT02** | 60,235,992 | 88.43% | 96.93% | 80.74% | 1.96% | 38.50 | 36.80% | 49.61 | 6541720 | 0.139 |
|  | **BT03** | 60,139,716 | 89.63% | 97.22% | 80.79% | 1.93% | 38.73 | 36.69% | 49.62 | 6541739 | 0.140 |
|  | **BT04** | 50,953,099 | 92.13% | 97.15% | 81.39% | 1.75% | 28.29 | 36.68% | 49.76 | 6540425 | 0.140 |
| **Melaka** | **TG01** | 60,055,409 | 89.75% | 94.96% | 78.38% | 1.94% | 37.74 | 36.64% | 49.51 | 6544485 | 0.141 |
|  | **TG02** | 60,229,934 | 90.25% | 96.51% | 79.94% | 1.90% | 38.58 | 36.61% | 49.59 | 6539651 | 0.140 |
|  | **TG03** | 60,084,921 | 89.26% | 92.86% | 76.46% | 1.96% | 36.70 | 36.73% | 49.49 | 6538065 | 0.135 |
|  | **TG04** | 60,145,433 | 89.23% | 96.67% | 79.45% | 2.05% | 38.43 | 36.76% | 49.41 | 6542046 | 0.141 |
|  | **JS01** | 60,122,408 | 88.77% | 96.93% | 80.86% | 1.91% | 38.38 | 36.59% | 49.58 | 6544293 | 0.143 |
|  | **JS02** | 60,207,361 | 88.54% | 96.90% | 80.38% | 1.99% | 38.46 | 36.64% | 49.55 | 6541870 | 0.141 |
|  | **JS03** | 60,155,275 | 89.22% | 97.16% | 80.85% | 1.82% | 38.62 | 36.78% | 49.62 | 6545219 | 0.140 |
|  | **JS04** | 60,112,867 | 89.30% | 97.08% | 80.94% | 1.87% | 38.34 | 36.83% | 49.41 | 6544087 | 0.141 |
| **Negeri Sembilan** | **AT01** | 60,131,538 | 95.25% | 97.77% | 82.69% | 1.34% | 39.79 | 36.60% | 49.79 | 6541917 | 0.142 |
|  | **AT02** | 60,114,987 | 95% | 97.41% | 81.71% | 1.48% | 39.78 | 36.72% | 49.98 | 6539802 | 0.139 |
|  | **AT03** | 60,103,580 | 94.66% | 97.71% | 81.88% | 1.54% | 39.89 | 36.68% | 49.89 | 6540903 | 0.139 |
|  | **AT04** | 60,107,642 | 94.71% | 97.28% | 80.24% | 1.67% | 39.58 | 36.72% | 49.72 | 6538835 | 0.141 |
|  | **TI01** | 60,073,536 | 95.11% | 97.93% | 82.40% | 1.44% | 39.90 | 36.43% | 49.83 | 6536911 | 0.139 |
|  | **TI02** | 60,203,747 | 94.15% | 97.52% | 79.10% | 1.77% | 39.80 | 36.08% | 49.72 | 6537436 | 0.140 |
|  | **TI03** | 60,128,683 | 95.88% | 98.06% | 86.00% | 0.93% | 39.64 | 37.00% | 49.76 | 6543137 | 0.144 |
|  | **TI04** | 60,059,857 | 95.17% | 97.64% | 82.99% | 1.38% | 39.73 | 36.62% | 49.83 | 6541103 | 0.142 |
|  | **TK02** | 60,164,831 | 95.18% | 97.34% | 81.71% | 1.48% | 39.53 | 36.56% | 49.66 | 6542395 | 0.144 |
|  | **TK03** | 60,149,845 | 94.89% | 97.38% | 81.08% | 1.60% | 39.76 | 36.86% | 49.80 | 6539784 | 0.141 |
|  | **TK04** | 48,079,370 | 94.65% | 97.44% | 79.95% | 1.78% | 29.03 | 36.69% | 50.06 | 6531060 | 0.134 |
|  | **TK05** | 58,671,466 | 94.08% | 97.63% | 80.27% | 1.78% | 39.02 | 36.49% | 49.91 | 6535918 | 0.138 |


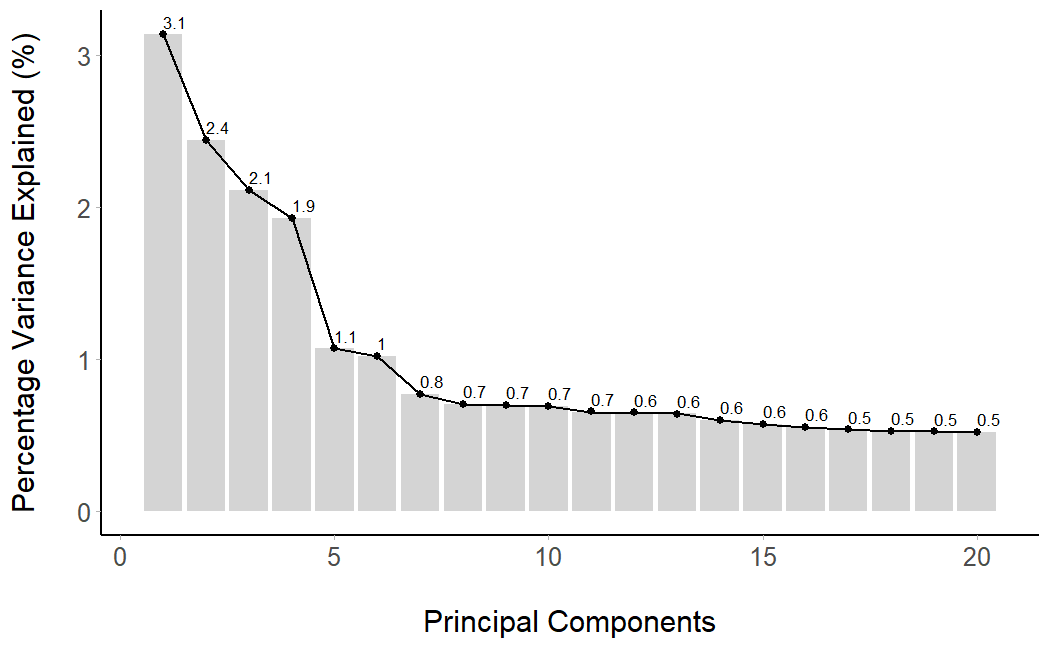


**Figure S 1. Percentage Variance Explained by Principal Components.**


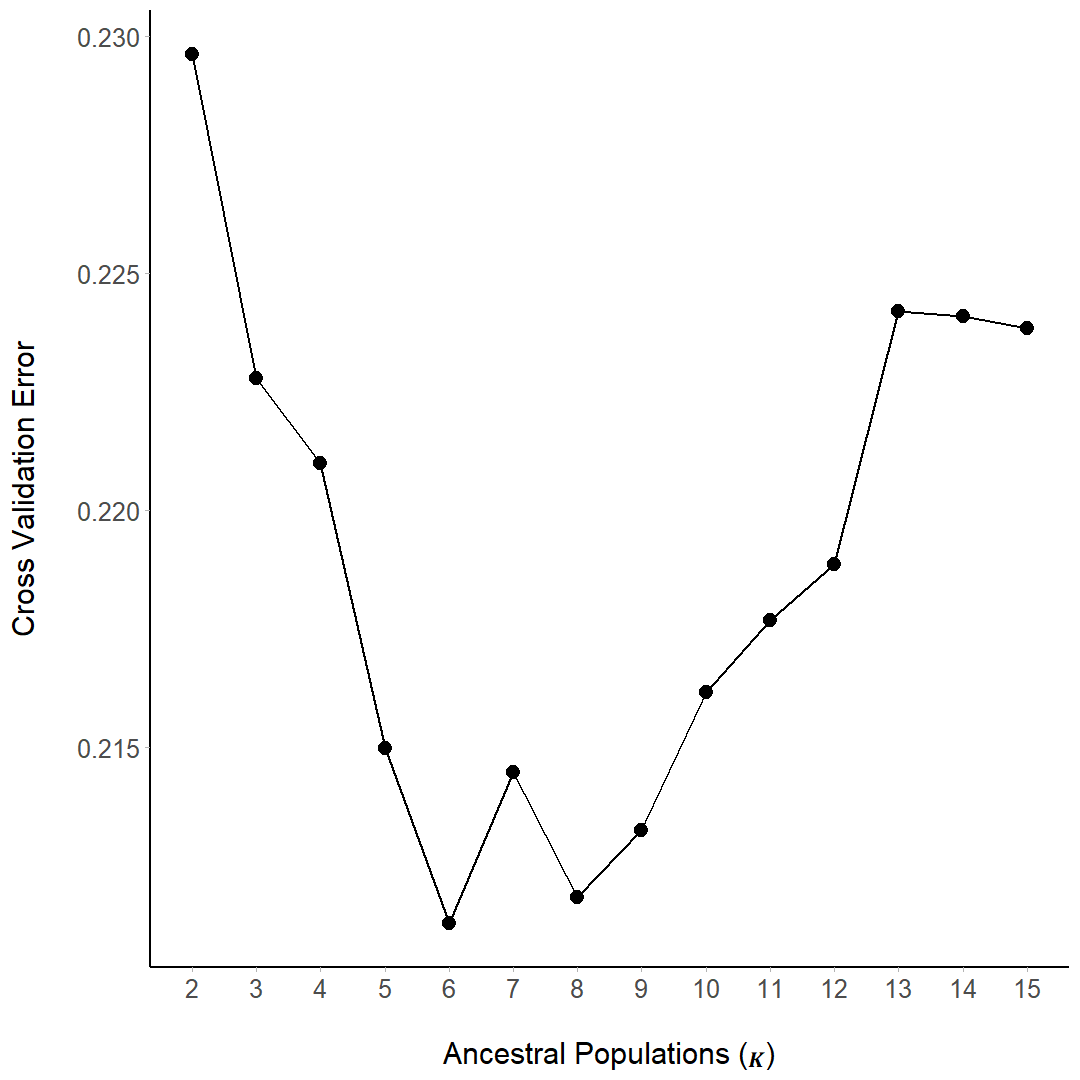


**Figure S2.** **Cross Validation Error Value for Different Number of Ancestral Populations (*K*) in ADMIXTURE Analysis.**


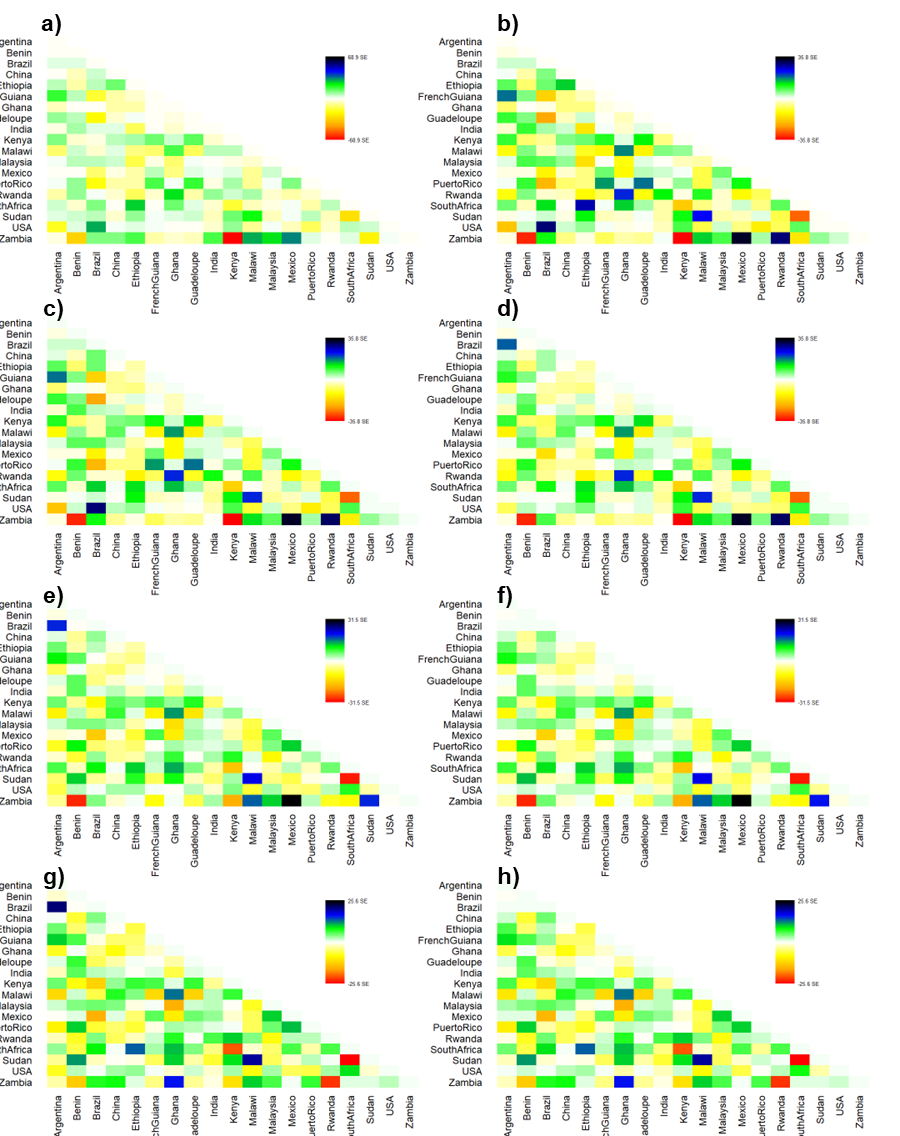


**Figure S3. Residual Analysis for TreeMix Plots.** (a) zero migration events; (b) one migration event; (c) two migration events; (d) three migration events; (e) four migration events; (f) five migration events; (g) six migration events; (h) seven migration events.


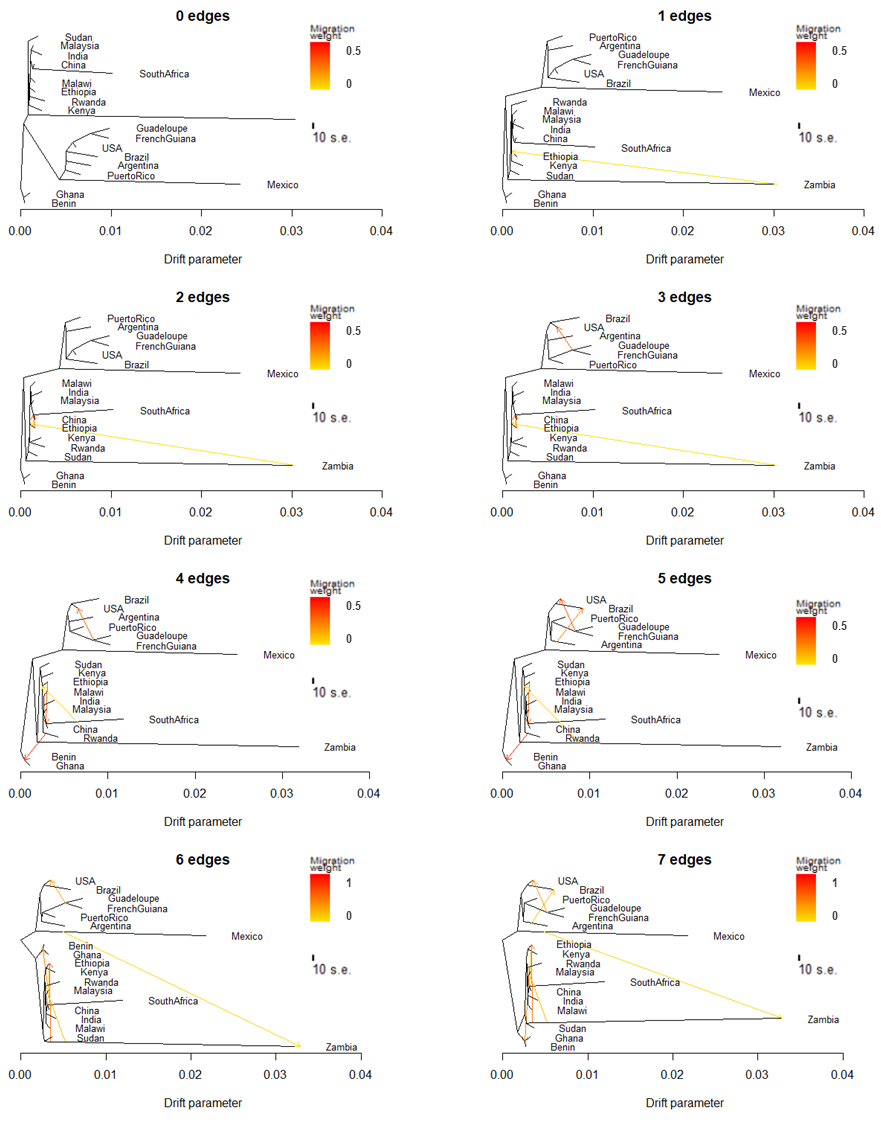


**Figure S4. TreeMix Plots for Migration Edges Ranging from None to Seven.**


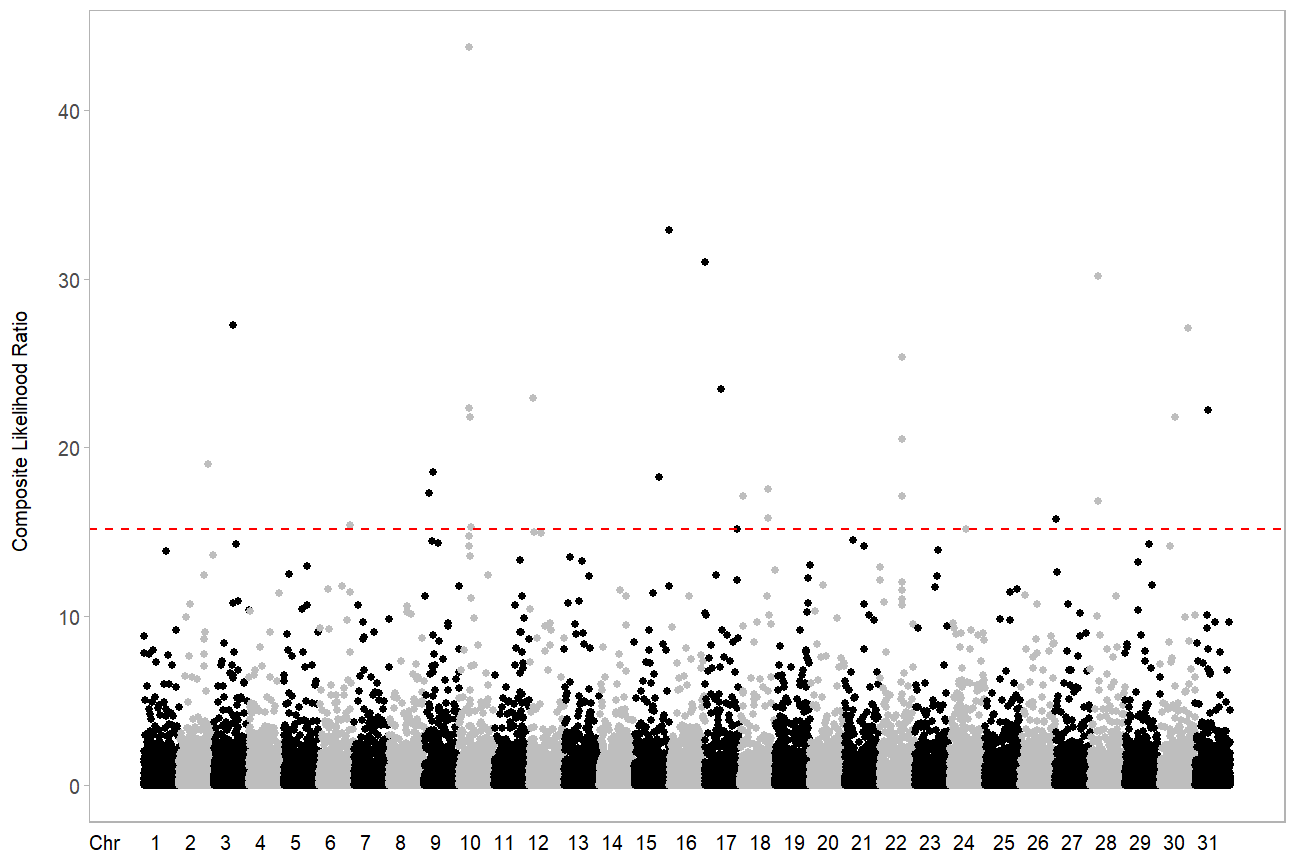


**Figure S5. Loci under positive selection in native fall armyworm (*S. frugiperda*) populations as determined by SweeD.** The y-axis represents the composite likelihood of being targeted by selective sweeps whereas the x-axis represents the chromosomes. Loci above the dashed red lines are among the top 0.1% regions being selected for.

**Table S3. Genes Undergoing Positive Selection in Fall Armyworm (*Spodoptera frugiperda*) Populations.** Loci are listed in descending order of composite likelihood ratio. Loci in bold are common to both ‘*All Invasive Populations*’ and the ‘*Malaysian Population Alone*’.

| **Chromosome** | **Loci** | **Protein** |
| --- | --- | --- |
| *MALAYSIAN POPULATION* | | |
| 19 | LOC126911854 | uncharacterised protein |
| **19** | **LOC118281122** | **BCL2/adenovirus E1B 19 kDa protein-interacting protein 3** |
| 19 | LOC126911831 | protein LSM12-like |
| 19 | LOC118280928 | intersectin-2-like |
| 19 | LOC118281208 | gelsolin, cytoplasmic-like |
| 19 | LOC126911827 | uncharacterised protein |
| 19 | LOC126911849 | uncharacterised protein |
| 19 | LOC118280931 | signal transducer and activator of transcription 5B |
| 19 | LOC126911857 | uncharacterised protein |
| 19 | LOC118280915 | monocarboxylate transporter 12-like |
| 19 | LOC118280917 | proteoglycan 4 |
| 19 | LOC126911812 | uncharacterised protein |
| 19 | LOC126911815 | zinc finger protein 16-like |
| 19 | LOC118281182 | zinc finger protein 26-like |
| 19 | LOC118281216 | DNA-directed RNA polymerase I subunit RPA49 |
| 19 | LOC126911798 | histone-lysine N-methyltransferase PRDM7-like |
| 19 | LOC118281209 | exopolyphosphatase PRUNE1 |
| 19 | LOC118280974 | uncharacterised protein |
| 19 | LOC118281247 | uncharacterised protein |
| **19** | **LOC118281183** | **solute carrier family 25 member 44** |
| 19 | LOC118280924 | uncharacterised protein |
| 19 | LOC118280866 | cytoplasmic FMR1-interacting protein-like |
| 29 | LOC118268282 | protein artichoke |
| **23** | **LOC118266783** | **rho GTPase-activating protein 23** |
| **23** | **LOC118267072** | **DNA repair protein RAD51 homolog 4** |
| 19 | LOC118281184 | bromodomain-containing protein 8 |
| 19 | LOC118281211 | zinc finger protein 62 |
| 19 | LOC126911900 | uncharacterised protein |
| **19** | **LOC118281268** | **cytoplasmic FMR1-interacting protein** |
| 19 | LOC118280870  LOC126911790  LOC126911791 | cytoplasmic FMR1-interacting protein-like |
| 19 | LOC126911843 | uncharacterised protein |
| 19 | LOC126911803 | protein PFC0760c-like |
| 19 | LOC126911822 | uncharacterised protein |
| 19 | LOC126911806 | zinc finger protein 83-like |
| 19 | LOC118280956 | zinc finger protein 878-like |
| 19 | LOC126911817 | E3 ubiquitin-protein ligase RNF220-like |
| 19 | LOC118280955 | copper chaperone for superoxide dismutase-like |
| 19 | LOC118280957 | zinc finger protein 135 |
| 19 | LOC118280982 | uncharacterised protein |
| 19 | LOC126911793 | uncharacterised protein |
| **19** | **LOC126911845** | **monocarboxylate transporter 12-like** |
| 19 | LOC118281887 | uncharacterised protein |
| **19** | **LOC126910632** | **signal transducer and activator of transcription 5B-like** |
| 19 | LOC126911794 | titin-like |
| 19 | LOC118281240 | uncharacterised protein |
| 19 | LOC126911808 | gelsolin, cytoplasmic-like |
| 19 | LOC126910624 | intersectin-2-like |
| 19 | LOC126911859 | uncharacterised protein |
| 19 | LOC118280930 | protein LSM12 |
| 19 | LOC126911867 | uncharacterised protein |
| 27 | LOC118263554 | cytochrome P450 4d2 |
| 4 | LOC118272929 | heparan sulfate 2-O-sulfotransferase pipe |
| 4 | LOC118272482 | uncharacterised protein |
| 4 | LOC118272481 | uncharacterised protein |
| 4 | LOC126910650 | uncharacterised protein |
| 4 | LOC126910649 | ethanolamine-phosphate cytidylyltransferase |
| 4 | LOC126910651 | meiotic recombination protein DMC1/LIM15 homolog |
| 4 | LOC118272870 | zinc finger CCCH domain-containing protein 18 |
| 4 | LOC118272486 | ribonucleases P/MRP protein subunit POP1 |
| 20 | LOC118281590 | probable serine/threonine-protein kinase DDB_G0267686 |
| 2 | LOC118269237 | uncharacterised protein |
| 8 | LOC118275813 | synaptosomal-associated protein 25 |
| 19 | LOC118280961 | ran GTPase-activating protein 1-like |
| 12 | LOC118262919 | uncharacterised protein |
| 12 | LOC118262822 | probable G-protein coupled receptor No18 |
| 6 | LOC118267421 | anaphase-promoting complex subunit 1 |
| 6 | LOC118267671  LOC118267772 | chymotrypsin-1-like |
| 6 | LOC126910755  LOC118267597 | chymotrypsin-2-like |
| *ALL INVASIVE POPULATIONS* | | |
| 18 | LOC118277859 | phosphatidylinositol 4-kinase type 2-beta |
| 18 | LOC118278096 | sarcoplasmic reticulum histidine-rich calcium-binding protein-like |
| 18 | LOC118278131 | uncharacterized protein |
| 18 | LOC118277874 | coiled-coil domain-containing protein R3HCC1L |
| 18 | LOC118278205 | polycomb group RING finger protein 3 |
| 18 | LOC118278171 | zinc finger protein 501-like |
| 18 | LOC118278094 | protein phosphatase methylesterase 1 |
| 18 | LOC118278101 | calcium uptake protein mitochondrial |
| 18 | LOC118278152 | uncharacterized protein |
| 18 | LOC118277873 | fibroblast growth factor 3 |
| 18 | LOC118278194 | MYG1 exonuclease |
| 18 | LOC118273720 | uncharacterized protein |
| 18 | LOC118277033 | uncharacterized protein |
| 18 | LOC126911727 | uncharacterized protein |
| 18 | LOC118264269 | uncharacterized protein |
| **19** | **LOC118281268** | **cytoplasmic FMR1-interacting protein** |
| 19 | LOC126911835 | calmodulin-like protein 4 |
| **19** | **LOC126911821** | **solute carrier family 25 member 44-like** |
| 19 | LOC118280957 | zinc finger protein 135 |
| 19 | LOC126911793 | uncharacterized protein |
| **19** | **LOC126911845** | **monocarboxylate transporter 12-like** |
| **19** | **LOC126910632** | **signal transducer and activator of transcription 5B-like** |
| **19** | **LOC126911830** | **BCL2/adenovirus E1B 19 kDa protein-interacting protein 3-like** |
| 19 | LOC118281008 | S-phase kinase-associated protein 2-like |
| 19 | LOC118281107  LOC118280863 | lysosomal alpha-mannosidase-like |
| 19 | LOC118280861 | mucin-17-like |
| 19 | LOC118280932 | igLON family member 5-like |
| **23** | **LOC118266783** | **rho GTPase-activating protein 23** |
| 23 | LOC118266782 | E3 ubiquitin-protein ligase TRIM37 |
| 23 | LOC118267074 | protoheme IX farnesyltransferase mitochondrial |
| **23** | **LOC118267072** | **DNA repair protein RAD51 homolog 4** |
| 19 | LOC118281287 | segmentation protein cap'n'collar |
| 12 | LOC118262558 | uncharacterized protein |
| 26 | LOC118264111 | phospholipid-transporting ATPase ABCA1 |
| 26 | LOC118264029 | protein yellow |
| 12 | LOC118262919 | uncharacterized protein |
| 21 | LOC126912043 | uncharacterized protein |
| 21 | LOC126912053 | uncharacterized protein |
| 21 | LOC118280495 | TBC1 domain family member 4 |
| 21 | LOC118280333 | dynein regulatory complex protein 11-like |
| 21 | LOC126912048 | uncharacterized protein |
| 21 | LOC118280212 | P protein |
| 21 | LOC118280211 | uncharacterized protein |
| 21 | LOC118280315 | uncharacterized protein |
| 12 | LOC118262778 | uncharacterized protein |
| 12 | LOC118262818 | uncharacterized protein |
| 12 | LOC118262823 | DNA topoisomerase 2-binding protein 1 |
| 12 | LOC118262897 | pre-rRNA-processing protein TSR2 homolog |
| 12 | LOC118263069 | alpha-1,2-mannosyltransferase ALG9 |
| 12 | LOC118263070 | tubulin beta chain-like |
| 18 | LOC118277904 | UDP-glycosyltransferase UGT5-like |
| 18 | LOC126911710 | uncharacterized protein |
| 18 | LOC118277900 | uncharacterized protein |
| 28 | LOC118264903 | CUGBP Elav-like family member 3-B |
